# Efficacy and safety of adjunctive Chinese herbal decoction in treating *Helicobacter pylori*–positive chronic atrophic gastritis: a real-world retrospective study

**DOI:** 10.3389/fmed.2025.1701915

**Published:** 2026-01-12

**Authors:** Jian Wang, Gang Chen, Dingting Zheng, Cuifang Ma

**Affiliations:** 1 Department of Gastroenterology, Jiaxing Second Hospital, Jiaxing, Zhejiang, China; 2 Department of Gastroenterology, Quzhou Hospital of Traditional Chinese Medicine, Quzhou, Zhejiang, China; 3 Department of Gastroenterology, The Second People's Hospital of Quzhou, Quzhou, Zhejiang, China; 4 Department of Infectious Diseases, The Quzhou Affiliated Hospital of Wenzhou Medical University, Quzhou People's Hospital, Quzhou, Zhejiang, China

**Keywords:** QYFD, chronic atrophic gastritis, *Helicobacter pylori*, clinical efficacy, adverse events

## Abstract

**Background:**

Chronic atrophic gastritis (CAG) associated with *Helicobacter pylori* (HP) infection is a common precancerous condition characterized by mucosal atrophy and gastrointestinal symptoms. Existing treatments show limited efficacy in symptom relief and are challenged by rising antibiotic resistance. Qingyoufang decoction (QYFD), a traditional Chinese herbal formula, is recommended for patients with spleen–stomach damp–heat syndrome (SSDHS), a common subtype of HP-positive CAG.

**Methods:**

This real-world retrospective study enrolled 113 patients with HP-positive CAG and SSDHS from a single hospital between September 2021 and September 2024. Patients received either standard bismuth-containing quadruple therapy (*n* = 44) or the same regimen plus QYFD (*n* = 69) for 2 weeks. Clinical efficacy was evaluated through HP eradication rates, SSDHS symptom scores, serum inflammatory markers (interleukin-6 [IL-6], interleukin-6 [IL-8], and tumor necrosis factor-alpha [TNF-*α*]), and 6-month symptom recurrence. Kaplan–Meier analysis and Cox regression analysis were used to assess prognostic factors.

**Results:**

The HP eradication rate was significantly higher in the QYFD group than in the control group (92.75% vs. 79.55%, *p* = 0.038). The overall symptom improvement rate (cure + marked improvement + improvement) was also higher in the QYFD group (92.75%) than in the control group (79.55%, *p* = 0.033). In an exploratory subgroup analysis (*n* = 8 control, *n* = 13 QYFD), inflammatory cytokines (IL-6, IL-8, TNF-*α*) decreased in both groups, with a greater trend toward reduction in the QYFD group (*p* < 0.05 for all). The 6-month symptom recurrence rate was lower in the QYFD group (15.87% vs. 37.14%, *p* = 0.017). The Cox regression analysis identified alcohol consumption (HR = 8.681, 95% CI: 1.070–70.413, *p* = 0.043) and severe atrophy (HR = 26.536, 95% CI: 3.390–207.735, *p* = 0.002) as independent risk factors for recurrence, while QYFD treatment was a protective factor (HR = 0.318, 95% CI: 0.107–0.840, *p* = 0.038).

**Conclusion:**

QYFD, as an adjunct to standard therapy, was associated with higher HP eradication rates, better symptom improvement, and lower symptom recurrence in patients with HP-positive CAG. A small exploratory subgroup suggested a potential reduction in inflammatory cytokines, which should be interpreted with caution. Further prospective studies are warranted to confirm these findings.

## Introduction

Chronic atrophic gastritis (CAG) is a challenging digestive disorder characterized by the atrophy of gastric mucosal glands, often associated with intestinal metaplasia or atypical hyperplasia (1, 2). In China, CAG is relatively prevalent, affecting approximately 20–30% of the population, with a rising incidence among younger individuals (3, 4). Although the pathogenesis of CAG is not fully understood, it is widely believed to be influenced by multiple factors, including genetic predispositions, environmental influences, pathogenic microbial infections, psychological status, and lifestyle choices (5). Among these, *Helicobacter pylori* (HP) infection is regarded as one of the most significant exogenous pathogenic factors (6). A meta-analysis conducted over the past decade revealed that HP-positive individuals had approximately 2.4-fold higher risk of developing CAG compared with HP-negative individuals (7). Furthermore, both HP infection and CAG are associated with an accelerated progression toward gastric cancer.

Currently, treatment for HP-positive CAG mainly relies on HP eradication therapy, gastric mucosal protectants, and proton-pump inhibitors, supplemented by lifestyle modifications such as dietary adjustment and stress management (8). However, these medications exhibit limited efficacy in these patients and may pose potential risks such as renal impairment, hypomagnesemia, and osteoporosis (9). More critically, the growing global crisis of antibiotic resistance in HP presents a formidable challenge. For instance, clarithromycin resistance has risen from 15.6% in the early 2000s to over 40% in 2020, while metronidazole resistance has increased from 58 to 78% during the same period (10). In response, contemporary clinical guidelines recommend bismuth-containing quadruple therapy as the first-line regimen, replacing the previously favored clarithromycin-based triple therapy (11). In cases of eradication failure, multiple rounds of different antibiotic combinations may be required. Nevertheless, prolonged antibiotic exposure can lead to gastrointestinal adverse effects, including diarrhea, appetite disturbances, nausea, and abdominal pain, as well as decreased drug sensitivity (12). Furthermore, the safety profile of antimicrobial therapy remains a significant concern in vulnerable populations—such as the elderly, children, and pregnant women—rendering such treatment approaches less feasible or inadvisable in these groups (13).

Effective management of patient symptoms is a critical factor in enhancing patient compliance. A recent study indicates that 56.7% of CAG patients experience one or more gastrointestinal symptoms, with dyspepsia and postprandial distress syndrome being the most prevalent, affecting over half of those symptomatic patients. Importantly, HP infection itself can cause gastrointestinal symptoms (14). However, conventional Western medical treatments—such as proton-pump inhibitors, gastric mucosal protectants, and prokinetic agents—have demonstrated limited efficacy in symptom relief. Therefore, there is an urgent need for innovative therapies, particularly non-antibiotic treatments, for HP-positive CAG patients to enhance HP eradication and alleviate associated symptoms.

Traditional Chinese medicine (TCM) has demonstrated significant success in personalizing treatment for CAG patients by considering their constitution, syndromes, and underlying etiology (15). Recently, the 2023 update of *the Expert Consensus on Traditional Chinese Medicine Diagnosis and Treatment of Chronic Gastritis* indicates that the spleen–stomach damp-heat syndrome (SSDHS) is the most prevalent symptom combination observed in HP-positive CAG patients (Table 1) (16). This syndrome comprises four primary symptoms: epigastric fullness, epigastric pain, heaviness of limbs, and loose stools, along with four secondary symptoms: poor appetite, a bitter taste in the mouth, halitosis, and fatigue. The guidelines advocate for adherence to the TCM principle of clearing heat and eliminating dampness. Qingyoufang, a traditional Chinese medicine decoction known for its heat-clearing and dampness-removing properties, is recommended for these patients (Table 2). Pharmacologically, QYFD combines heat-clearing and anti-inflammatory herbs (e.g., *Scutellaria baicalensis* and *Coptis chinensis*), dampness-resolving and motility-regulating herbs (e.g., *Magnolia officinalis*), and spleen-fortifying and mucosal-protective herbs (e.g., *Atractylodes macrocephala* and *Codonopsis pilosula*), forming a coherent adjunct to quadruple therapy for HP-positive CAG. Consequently, this study aimed to analyze the clinical efficacy and safety of Qingyoufang decoction (QYFD) in HP-positive CAG patients.

**Table 1:** Manifestations and diagnostic criteria of SSDHS.

Symptom	None (0)	Mild	Moderate	Severe	Score
Epigastric fullness*	No sensation of fullness.	Occasional upper-abdominal fullness after meals, lasting < 1 h.	Noticeable fullness or post-meal bloating, each episode lasting 1–3 h.	Persistent, severe fullness lasting more than 3 h per episode.	
Epigastric pain*	No pain.	Occasional pain (dull, distending, or stabbing) resolving within 1 h.	Frequent pain (dull, distending, or stabbing), tolerable, lasting 1–3 h.	Intense abdominal pain (dull/distending/stabbing), intolerable, lasting > 3 h.	
Heaviness of limbs*	No sensation of heaviness.	Occasional limb heaviness resolves within 1 h.	Daily heaviness with mild impact on work, resolves in 2–3 h.	Continuous heaviness, significantly impairs work, not relieved by rest.	
Loose stools*	Normal stool consistency.	Soft or slightly loose stools, ≤ 3 bowel movements/day.	Loose stools, 4–5 bowel movements/day.	Mushy stools, > 6 bowel movements/day.	
Poor appetite^#^	Normal appetite.	Decreased interest in eating but maintains usual intake.	Little or no appetite; food intake reduced by ~ 1/3.	Anorexia; intake reduced by > 1/2.	
Bitter taste in mouth^#^	No bitter or sticky taste.	Occasional bitter/sticky sensation, no impact on eating.	Frequent bitter/sticky sensation, mildly affects eating.	Persistent bitter/sticky taste, significantly impairs eating.	
Halitosis (bad breath)^#^	No noticeable odor.	Subjective awareness of bad breath.	Bad breath noticeable to others.	Severe, offensively strong halitosis.	
Fatigue^#^	Normal energy level.	Mild fatigue, less talkative but able to work.	Noticeable fatigue, reduced speech and work capacity.	Extreme fatigue, strong desire to lie down; markedly impaired work ability.	

* = Main symptom, scoring: none = 0; mild = 2; moderate = 4; severe = 6; # = secondary symptom, scoring: none = 0; mild = 2; moderate = 4; severe = 6; diagnostic criteria for spleen–stomach damp-heat syndrome: at least two main symptoms (*) and two secondary symptoms (#) must be present concurrently.

**Table 2:** Composition of the QYFD used in this study.

No.	Herb name (English)	Herb name (Pinyin)	Dosage (g)
1	Codonopsis Root	Dang Shen	15
2	Lindera Root	Wu Yao	10
3	Bletilla Rhizome	Bai Ji	6
4	Atractylodes Rhizome	Bai Zhu (Fu-chao, bran-fried)	12
5	Poria	Fu Ling	12
6	Licorice Root	Zhi Gan Cao	5
7	Acorus Rhizome	Shi Chang Pu	12
8	Scutellaria Root	Huang Qin	10
9	Coptis Rhizome	Huang Lian	5
10	Magnolia Bark	Hou Po	10
11	Salvia Root	Dan Shen	15
12	Barley Sprout	Mai Ya (Chao, fried)	15

## Methods

### Study design

This study retrieved clinical data from patients with HP-positive CAG who visited the outpatient clinic or were hospitalized at Quzhou Traditional Chinese Medicine Hospital between September 2021 and September 2024. Patients included in this retrospective real-world study met the following criteria: (1) confirmed HP-positive status through a ^13^C-urea breath test analyzer or pathological examination; (2) pathological diagnosis of CAG; (3) diagnostic criteria for SSDHS were based on a standardized TCM symptom scoring system (Supplementary Table S2). The syndrome includes four primary symptoms (epigastric fullness, epigastric pain, heaviness of limbs, and loose stools) and four secondary symptoms (poor appetite, bitter taste in the mouth, halitosis, and fatigue). A diagnosis of SSDHS was confirmed when at least two primary symptoms and two secondary symptoms were present concurrently; (4) aged between 20 and 75 years; and (5) no use of acid-suppressing drugs or antibiotics within 28 days prior to enrollment. The exclusion criteria were as follows: (1) presence of other gastrointestinal diseases such as reflux esophagitis or gastrointestinal malignancies; (2) previous history of upper gastrointestinal surgery; (3) serious medical conditions such as acute liver or kidney failure or acute myocardial infarction; (4) known allergies to any drugs or antibiotics used in this study; and (5) patients who are preparing for pregnancy or are pregnant during the study period. This study received approval from the Ethics Committee of Quzhou Traditional Chinese Medicine Hospital, and all investigations were conducted in accordance with the Declaration of Helsinki (revised in 2013).

### Treatment

Patients in the Western medicine group (control group) were treated for 2 weeks with a bismuth-containing quadruple regimen comprising esomeprazole enteric-coated tablets (0.02 g per dose), colloidal pectin–bismuth capsules (0.2 g per dose), amoxicillin capsules (1.0 g per dose), and furazolidone tablets (0.1 g per dose), each administered twice daily 30 min after breakfast and dinner.

Patients in the combined Chinese–Western medicine group (experimental group) received the identical quadruple regimen supplemented with QYFD—a standardized traditional Chinese medicine decoction (see Table 2 for formula details). The decoction was dispensed under sterile conditions in 200-mL bags by the Decoction Room of Quzhou Traditional Chinese Medicine Hospital and administered warm 30 min after breakfast for the same 2-week period.

### Data collection and outcomes evaluations

1. General information: The patient’s gender, age, course of disease, endoscopic pathological results (atrophy and intestinal metaplasia), living habits, and common diseases were collected.
2. Modern medicine observation indicators: HP eradication rate: The ^13^C-urea breath test was repeated 4 weeks after drug withdrawal, and a delta over baseline (DOB) value < 4 showed that HP was considered eradicated. Inflammation level: 5 mL of fasting venous blood was collected from each patient before and after the treatment, and the levels of IL-6, IL-8, and TNF-*α* in serum were detected using enzyme-linked immunosorbent assay (ELISA) kits.
3. Traditional Chinese medicine observation indicators: The symptoms of CAG in patients were quantitatively scored before and after the treatment, following *the Guiding Principles for Clinical Research of New Chinese Medicines*. As shown in Table 1, the main and secondary symptoms of SSDHS were graded and scored using an integral method. Symptoms were categorized into four levels: none, mild, moderate, and severe, with main symptoms scored as 0, 2, 4, and 6 points and secondary symptoms scored as 0, 1, 2, and 3 points. Recovery was defined as a decrease of ≥ 95% in the total score after the treatment; marked improvement was defined as a decrease of 70–95%; effectiveness was defined as a decrease of 30–70%; and ineffectiveness was defined as a decrease of less than 30%.
4. Follow-up within 6 months: After the treatment concluded, a 6-month follow-up was conducted through a review of the hospitalization and outpatient follow-up records of the two groups of patients who exhibited improved symptoms (including recovery, marked improvement, and overall effectiveness). The recurrence or aggravation of clinical symptoms, indicated by an increase in the total score of traditional Chinese medicine (TCM) syndromes compared to the post-treatment assessment, was recorded as a positive endpoint event. Conversely, if no endpoint event occurred within the 6 months, it was classified as a cutoff event.

### Statistical analysis

The normality of continuous variables was tested using the Shapiro–Wilk test. Normally distributed data were analyzed using the independent-samples t-test, whereas non-normally distributed data were analyzed using the Mann–Whitney U-test. Continuous variables were summarized as mean ± SD or median (P25, P75). Between-group differences were tested using independent t-tests (Welch’s if variances were unequal) or Mann–Whitney U-tests; within-group (pre–post) differences were tested using paired t-tests or Wilcoxon signed-rank. Categorical data were shown as percentages and analyzed using chi-squared, continuity-corrected chi-squared, or Fisher’s exact test. Ordinal data were assessed using the rank-sum test. Kaplan–Meier curves with log-rank tests were used for recurrence analysis. Cox regression analysis was performed to identify associated risk factors, with *p* < 0.05 considered statistically significant. The proportional hazards assumption was tested using Schoenfeld residuals. All statistical analyses were conducted using SPSS version 26.0 (IBM Corp., USA). A two-tailed test was applied for all analyses, with a significance level of *α* = 0.05.

## Results

### Patient characteristics and outcomes

**Figure 1:**
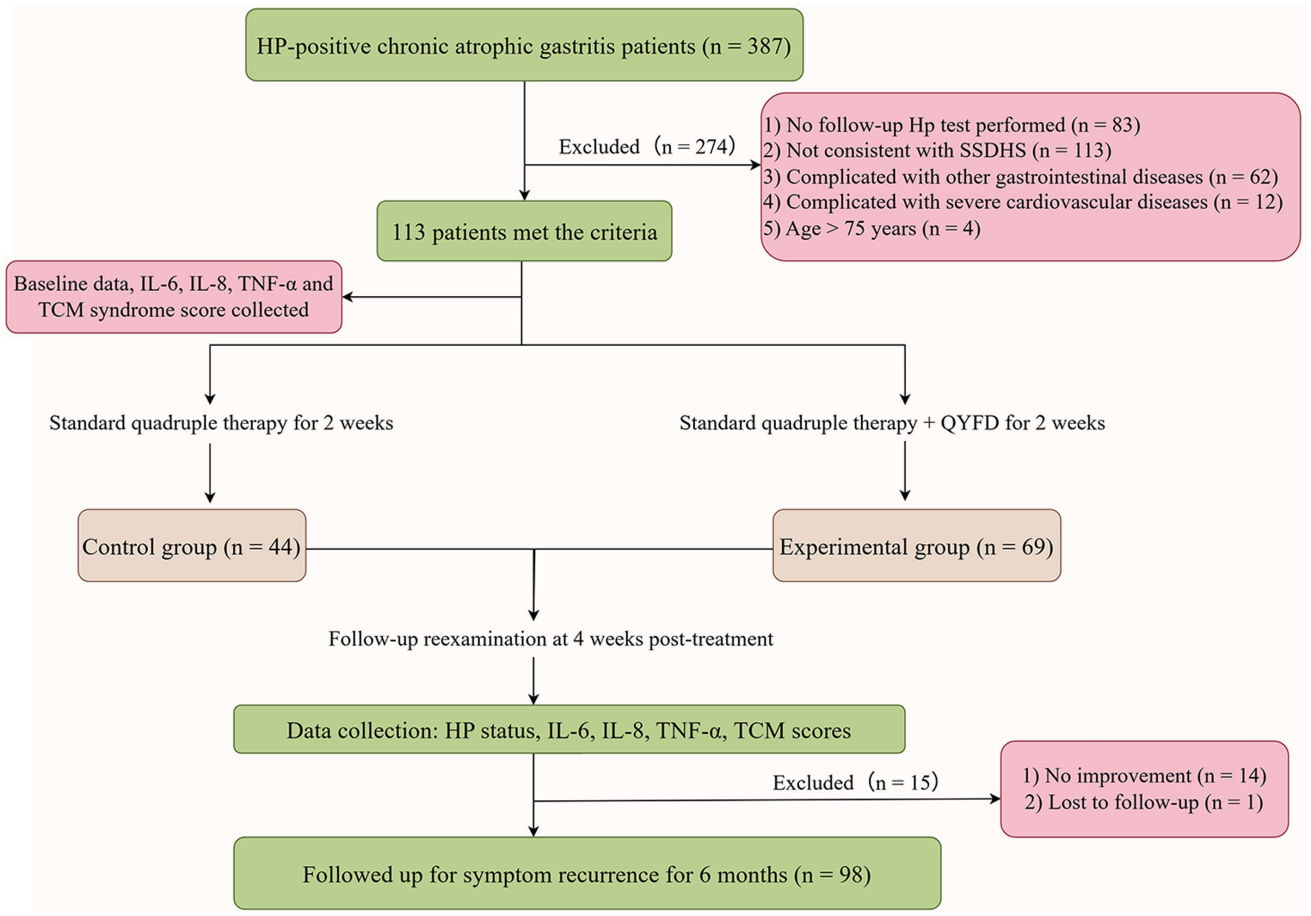
Study flow diagram.

This study retrospectively analyzed 387 patients diagnosed with HP-positive CAG who were admitted to Quzhou Hospital of Traditional Chinese Medicine between September 2021 and September 2024. A total of 113 patients met the inclusion criteria, with 44 receiving conventional quadruple therapy and 69 receiving a combination of QYFD and quadruple therapy. Figure 1 illustrates the detailed patient enrollment process. No statistically significant differences were observed between the two groups regarding clinical characteristics, including age, gender, disease duration, gastric mucosal atrophy, and intestinal metaplasia. Specific patient characteristics are summarized in Table 3.

**Table 3:** Baseline demographic and clinical characteristics of study participants.

Groups	Control group (*n* = 44)	Experimental group (*n* = 69)	*X^2^/t*	*p*
Gender, *n* (%)			0.183	0.669
Male	25 (56.81)	42 (60.87)		
Female	19 (43.19)	27 (39.13)		
Age (years, mean ± *SD*)	59.54 ± 6.82	60.33 ± 5.52	0.645	0.521
Duration of illness (years, mean ± SD)	3.86 ± 1.48	4.07 ± 1.40	0.751	0.455
Gastric atrophy, *n* (%)			0.252	0.882
Mild	12 (27.28)	20 (28.99)		
Moderate	22 (50.00)	36 (52.17)		
Severe	10 (22.72)	13 (18.84)		
Intestinal metaplasia, *n* (%)			2.834	0.418
None	22 (50.00)	35 (50.72)		
Mild	11 (25.00)	18 (26.09)		
Moderate	10 (22.72)	10 (14.50)		
Severe	1 (2.28)	6 (8.69)		
Smoking, *n* (%)	22 (50.00)	36 (52.17)	0.051	0.822
Alcohol consumption, *n* (%)	20 (45.45)	38 (55.07)	0.995	0.319
Hypertension, *n* (%)	9 (20.45)	12 (17.39)	0.167	0.683
Diabetes mellitus, *n* (%)	4 (9.09)	8 (11.59)	0.012	0.914

### HP eradication rates and TCM therapeutic efficacy

Four weeks after treatment withdrawal, HP status was reassessed using the ^13^C-urea breath test. The HP eradication rate was 92.75% (64/69) in the experimental group and 79.55% (35/44) in the control group, with a statistically significant difference between the two groups (*p* = 0.038) (Table 4).

**Table 4:** Comparison of HP eradication rates and TCM therapeutic efficacy between the two groups.

Groups	Control group (*n* = 44)	Experimental group (*n* = 69)	*X^2^/Z*	*p*
HP eradication rates, *n* (%)			4.318	0.038
Negative	35 (79.55)	64 (92.75)		
Positive	9 (20.45)	5 (7.25)		
TCM therapeutic efficacy, *n* (%)			2.137	0.033
Ineffectiveness	9 (20.45)	5 (7.25)		
Effectiveness	17 (38.64)	20 (28.99)		
Markedly effective	10 (22.73)	28 (40.58)		
Recovery	8 (18.18)	16 (23.18)		

SSDHS-related symptom scores before and after the treatment are presented in Supplementary Table S1. Both groups showed reductions in symptom scores following treatment across all listed domains. Intra-group comparisons indicated improvements in each symptom, while between-group comparisons of post-treatment values revealed significantly lower scores in the experimental group for epigastric fullness, epigastric pain, heaviness of limbs, and fatigue.

The distribution of TCM therapeutic efficacy outcomes is summarized in Table 4. In the experimental group, 23.18% (16/69) of patients achieved recovery, 40.58% (28/69) were markedly improved, 28.99% (20/69) were effective, and 7.25% (5/69) were ineffective. In contrast, the corresponding figures in the control group were 18.18% (8/44), 22.73% (10/44), 38.64% (17/44), and 20.45% (9/44), respectively. The difference in efficacy distribution between the groups was statistically significant (*p* = 0.033).

### Levels of inflammatory factors

Serum IL-6, IL-8, and TNF-*α* levels were measured in 8 patients from the control group and 13 patients from the experimental group before and after the treatment (Table 5). Baseline levels of IL-6 (22.97 ± 3.01 vs. 23.74 ± 1.99 pg/mL), IL-8 (49.06 ± 4.72 vs. 50.30 ± 5.01 pg/mL), and TNF-*α* (35.35 ± 3.43 vs. 33.98 ± 3.22 pg/mL) showed no significant differences between the groups (*p* > 0.05). After the treatment, IL-6 levels decreased to 19.50 ± 2.23 pg/mL in the control group and 14.36 ± 2.32 pg/mL in the experimental group (*t* = 6.748, *p* < 0.001). IL-8 levels declined to 43.78 ± 3.65 and 38.27 ± 3.43 pg/mL, respectively (*t* = 5.189, *p* < 0.001). TNF-α levels were reduced to 29.10 ± 3.04 and 22.82 ± 2.92 pg./mL, respectively (*t* = 5.025, *p* < 0.001).

**Table 5:** Comparison of serum inflammatory factor levels (*x* ± s, pg/mL) before and after treatment.

Groups	Control group (*n* = 8)	Experimental group (*n* = 13)	*t*	*p*
IL-6				
Before treatment	22.97 ± 3.01	23.74 ± 1.99	0.643	0.533
After treatment	19.50 ± 2.23	14.36 ± 2.32	5.054	<0.001
IL-8				
Before treatment	49.06 ± 4.72	50.30 ± 5.01	0.571	0.575
After treatment	43.78 ± 3.65	38.27 ± 3.43	3.435	0.004
TNF-α				
Before treatment	35.35 ± 3.43	33.98 ± 3.22	0.910	0.388
After treatment	29.10 ± 3.04	22.82 ± 2.92	4.664	<0.001

### SSDHS-related symptom recurrence

Recurrence analysis was performed only among patients who achieved recovery, marked improvement, or improvement after the treatment (35 in the control group and 63 in the QYFD group). Patients with ineffective responses were not eligible for recurrence assessment, as recurrence can only occur after an initial response.

As shown in Table 6, recurrence occurred in 13 patients (37.14%) in the control group and 10 patients (15.87%) in the experimental group. The difference in recurrence rates between the groups was statistically significant (*p* = 0.017).

**Table 6:** Comparison of 6-month symptom recurrence between the two groups.

Groups	Recurrence (*n*, %)	No recurrence (*n*, %)	*X^2^*	*p*
Control group (*n* = 35)	13 (37.14)	22 (62.86)	5.667	0.017
Experimental group (*n* = 63)	10 (15.87)	53 (84.13)		

**Figure 2:**
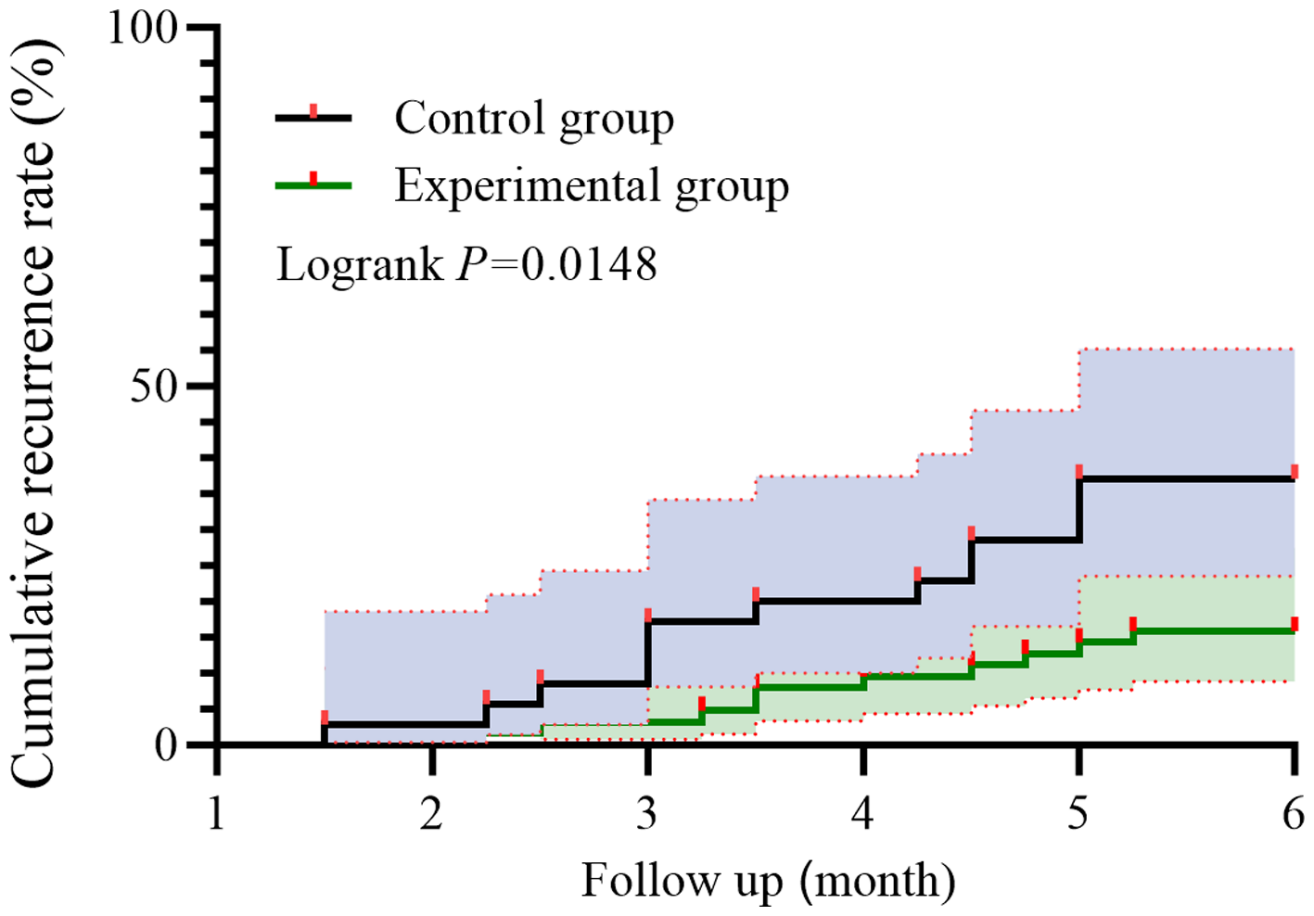
Kaplan–Meier curves of symptom recurrence in patients with HP-positive CAG. Red markers denote recurrence events; Individuals without recurrence by 6 months were censored at 6 months.

Kaplan–Meier survival analysis revealed a lower cumulative recurrence rate in the experimental group compared to the control group over the 6-month follow-up period. As shown in Figure 2, the difference between the two curves was significant based on the log-rank test (*p* = 0.0148).

### Prognostic factors for SSDHS-related symptoms

**Figure 3:**
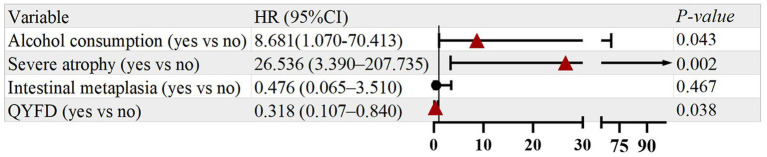
Univariate analysis of prognostic factors for symptom recurrence.

As shown in Figure 3, univariate Cox regression analysis identified that alcohol consumption (yes vs. no; HR = 19.897, 95% CI: 2.614–151.466, *p* = 0.004), severe atrophy (yes vs. no; HR = 26.524, 95% CI: 7.370–95.455, *p* < 0.001), and intestinal metaplasia (yes vs. no; HR = 5.550, 95% CI: 1.564–19.691, *p* = 0.008) were potential risk factors for the recurrence of SSDHS-related symptoms. In addition, the use of QYFD (yes vs. no) was associated with a reduced risk of recurrence (HR = 0.364, 95% CI: 0.129–1.023, *p* = 0.055).

**Figure 4:**
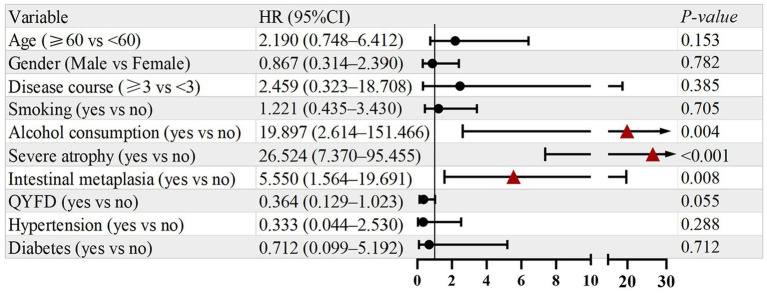
Multivariate analysis of prognostic factors for symptom recurrence.

As presented in Figure 4, variables with *p* < 0.1 in the univariate analysis were subsequently included in the multivariate Cox regression model. The results showed that alcohol consumption (HR = 8.681, 95% CI: 1.070–70.413, *p* = 0.043) and severe atrophy (HR = 26.536, 95% CI: 3.390–207.735, *p* = 0.002) remained independent risk factors. Meanwhile, treatment with QYFD was independently associated with a significantly lower risk of recurrence (HR = 0.318, 95% CI: 0.107–0.840, *p* = 0.038). The proportional hazards assumption was satisfied for all models.

### Adverse events

During the treatment period, one patient in the control group experienced dizziness, and two patients experienced nausea, resulting in a total adverse event rate of 6.8%. In contrast, two patients in the experimental group experienced nausea, leading to a total adverse event rate of 2.9%. Post-treatment evaluations revealed no abnormalities in the blood, urine, stool, routine, or liver and kidney function tests for all patients, indicating that QYFD has a favorable safety profile.

## Discussion

In this real-world retrospective study, we focused on HP-positive CAG, a patient subgroup characterized by a high prevalence and severity of gastrointestinal symptoms. Our analyses indicated that adding QYFD to standard quadruple therapy was associated with greater symptom improvement, higher HP eradication rates, and a reduced risk of symptom recurrence. An exploratory subgroup with a small sample size suggested a potential trend toward lower levels of inflammatory markers, but this observation should be interpreted with caution. These findings underscore the therapeutic value of syndrome-specific Chinese herbal interventions in managing HP-CAG, providing real-world evidence to support the integration of traditional medicine into modern clinical practice for digestive diseases.

TCM has been increasingly adopted as a non-antibiotic adjunctive approach for managing CAG and HP infection. Previous studies have demonstrated that several classical herbal formulas—including Banxia Xiexin decoction (17, 18), Moluo Dan (19), Weierkang pills (20), and Liujunzi decoction (21)—can enhance HP eradication, slow the progression of gastric mucosal atrophy, and effectively alleviate clinical symptoms. According to modern TCM theory, CAG is mainly characterized by spleen–stomach deficiency, where a hypoacidic gastric environment facilitates HP colonization and persistence. To address this pathological condition, QYFD follows the therapeutic principle of strengthening the spleen and clearing internal heat. Comprising multiple medicinal herbs, it exerts synergistic, multi-target, and multi-pathway actions that help restore physiological balance and enhance host defense. Polysaccharides from *Codonopsis pilosula* have been shown to alleviate spleen deficiency-related symptoms by modulating pro-inflammatory cytokines and maintaining gut microbiota homeostasis (22). In addition, active compounds such as flavonoids, saponins, alkaloids, and steroids present in Codonopsis exhibit immunomodulatory, antimicrobial, and antitumor properties (23, 24). Coptis chinensis has shown notable efficacy against HP, with berberine identified as its principal bioactive constituent. Berberine is believed to inhibit bacterial metabolism by blocking glucose oxidation and related intermediates and also to exert anti-inflammatory effects via the Toll-like receptor 4/nuclear factor kappa B (NF-κB) signaling pathway (25, 26). Additionally, berberine and Codonopsis polysaccharides have been reported to downregulate gastric mucosal pro-inflammatory cytokines (IL-6, IL-8, TNF-*α*), indicating immuno-inflammatory modulation (27, 28). Poria polysaccharides inhibit JAK2 and STAT3 phosphorylation, thereby improving intestinal barrier integrity and mitigating inflammation (29). Moreover, they promote the secretion of immune-stimulating mediators while suppressing immunosuppressive factors, ultimately enhancing host immune responses (30). Extracts of *Acorus calamus* rhizome and its bioactive components have been reported to exert antibacterial effects by modulating cell membrane permeability and lipid composition (31). These findings suggest that QYFD may contribute to restoring spleen–stomach function and immune homeostasis, reducing gastric mucosal injury, suppressing the excessive release of inflammatory mediators, and interfering with HP colonization—thereby potentially improving disease outcomes and supporting long-term management of CAG.

In China, bismuth-containing quadruple regimens based on furazolidone and amoxicillin are widely used in clinical practice due to their relatively low resistance rates and cost-effectiveness. Zhang et al. conducted a retrospective analysis of 992 patients with HP infection treated at the Second Affiliated Hospital of Zhejiang University in 2015, reporting that this regimen achieved an eradication rate of nearly 95%, with manageable side effects (32). However, subsequent data from the same hospital between 2017 and 2020, involving 16,784 patients, revealed a notable decline in efficacy, with the eradication rate decreasing to 87.6% (14,707 cases) (33). More recently, a real-world study published in 2024 further reported a reduced eradication rate of approximately 84.4% with the same regimen. These findings suggest a downward trend in the efficacy of this standard antibiotic-based therapy. In our study, the eradication rate in the control group, which received the same regimen, was only 79.55%. This may be attributed to increased antibiotic resistance and atrophy of gastric mucosal glands in the study population (34, 35). Notably, adding QYFD to the standard regimen significantly improved the eradication rate to 92.75% while maintaining good safety and tolerability. Against the backdrop of escalating antibiotic resistance, non-antibiotic adjunctive therapies such as QYFD and other traditional Chinese medicine-based approaches may offer promising clinical potential for enhancing HP eradication outcomes.

The eradication of HP significantly impacts symptom relief. A recent meta-analysis of 29 randomized controlled trials involving 6,781 HP-positive patients with functional dyspepsia demonstrated that successful HP eradication was significantly associated with symptom improvement compared to failed eradication (RR = 0.65, 95% CI: 0.52–0.82) (36). Consistently, in our study, patients who achieved HP clearance experienced varying degrees of symptom alleviation, while those who remained HP-positive after treatment reported persistent symptoms. However, HP-positive CAG presents a more complex clinical picture. In addition to HP-induced discomfort, symptoms may arise from the underlying mucosal atrophy and impaired gastric barrier and motility. Previous studies have shown that approximately 24% of CAG patients experience heartburn, 12% report reflux, and other common symptoms include postprandial fullness (7.1%) and early satiety (10.1%) (37). Currently, symptom management in CAG primarily relies on proton-pump inhibitors (PPIs) and mucosal protective agents. Nevertheless, their efficacy is limited in patients with recurrent symptoms. More importantly, prolonged PPI use has been associated with adverse outcomes such as gastric cancer, neuroendocrine tumors, chronic kidney dis*e*ase, and heart failure and may even exacerbate mucosal atrophy (34, 38, 39). In this context, our results showed that among patients with successful HP eradication, those receiving QYFD treatment achieved significantly better symptom control than controls, suggesting its potential advantage in symptom relief. While existing predictive models have largely focused on assessing the risk of intestinal metaplasia, dysplasia, or psychological comorbidities in CAG, few have addressed symptom recurrence or persistence. Interestingly*, a prior* Mendelian randomization study has established a link between functional dyspepsia and alcohol consumption (40). Furthermore, the severity of mucosal atrophy may influence the efficacy of prokinetic agents, such as acotiamide, suggesting a physiological basis for the association between gastric mucosal status and symptom severity (41). For instance, both antral and corporal atrophies have been linked to delayed gastric emptying and impaired motility, which may contribute to symptoms such as epigastric fullness and discomfort. These findings partially support our observation that alcohol intake and severe mucosal atrophy were associated with symptom recurrence in this cohort.

This study has several limitations. First, due to the limited sample size and single-center retrospective design, the results may be subject to bias, and the relatively small number of outcome events may have reduced the precision of estimates (as reflected by wide confidence intervals in the Cox regression) and increased the risk of model overfitting, although the proportional hazards assumption was satisfied. Moreover, as a real-world retrospective study, treatment adherence could not be strictly controlled, and a small number of patients were lost to follow-up during the 6-month observation period. Second, serum inflammatory markers were not available for all patients, and HP antibiotic susceptibility was not assessed, which may have introduced additional bias. Additionally, some studies suggest that probiotics may enhance eradication when added to quadruple therapy, but probiotics were not routinely used at our center and were not evaluated in this study. The complexity of symptom recurrence renders the results susceptible to confounding factors such as immune status, HP subtypes, antibiotic sensitivity, lifestyle, and psychological stress, and the lack of a unified quantitative standard for Traditional Chinese Medicine symptom assessment may affect diagnostic consistency. Moreover, because the assessors were aware of treatment allocation, unconscious inclination toward the experimental group could not be entirely ruled out. Furthermore, the 6-month follow-up duration may be insufficient to fully capture long-term recurrence in CAG, and longer follow-up (12–24 months) would be warranted to assess sustained outcomes more comprehensively. Future research should aim to expand the sample size and adopt multicenter and prospective designs to validate these findings.

## Conclusion

This real-world retrospective study examined patients with HP-positive CAG, a population at increased risk for gastrointestinal symptoms and persistent HP infection. Our analyses showed that adjunctive treatment with the traditional Chinese herbal decoction Qingyoufang (QYFD) was associated with better symptom control, higher HP eradication rates, and a lower rate of symptom recurrence compared with standard therapy alone. An exploratory analysis based on a small subgroup suggested that QYFD might be associated with reductions in pro-inflammatory cytokines (IL-6, IL-8, and TNF-*α*), indicating a potential but preliminary trend toward modulation of gastric mucosal inflammation. These findings suggest that QYFD may represent a promising non-antibiotic complementary approach for managing HP-positive CAG, particularly in patients with spleen–stomach damp–heat syndrome, although further prospective validation is needed.

## Data Availability

The original contributions presented in the study are included in the article/Supplementary material, further inquiries can be directed to the corresponding author.
