# Supplementary material for: Efficacy and safety of adjunctive Chinese herbal decoction in treating Helicobacter pylori–positive chronic atrophic gastritis: a real-world retrospective study
Source: Front Med (Lausanne). 2026 Jan 12;12:1701915. doi: 10.3389/fmed.2025.1701915 (PMC12833376; doi:10.3389/fmed.2025.1701915)
Supplement: Supplementary file 1 [file Supplementary_file_1.docx]

Table S1. Comparison of SSDHS-Related Symptom Scores [M (P25, P75)]

| **Symptom** | Control group | | |  | Experimental group | | |  |
| --- | --- | --- | --- | --- | --- | --- | --- | --- |
|  | **n** | **Before Treatment** | **After Treatment** | **Change from baseline Δ** | **n** | **Before Treatment** | **After Treatment** | **Change from baseline Δ** |
| Epigastric fullness | 41 | 2 (2.00, 2.00) | 2 (0.00, 2.00)^*^ | 0 (0.00, 2.00) | 54 | 2 (2.00, 2.00) | 0 (0.00, 2.00)^*#^ | 2 (0.00, 2.00) |
| Epigastric pain | 32 | 2 (2.00, 2.00) | 0 (0.00, 2.00)^*^ | 2 (0.00, 2.00) | 49 | 2 (2.00, 2.00) | 0 (0.00, 2.00)^*#^ | 2 (0.00, 2.00) |
| Loose stool | 28 | 2 (2.00, 2.00) | 0 (0.00, 2.00)^*^ | 2 (0.00, 2.00) | 44 | 2 (2.00, 2.00) | 0 (0.00, 2.00)^*^ | 2 (0.00, 2.00) |
| Heaviness of limbs | 29 | 2 (2.00, 2.00) | 2 (0.00, 2.00)^*^ | 0 (0.00, 2.00) | 34 | 2 (2.00, 2.00) | 0 (0.00, 2.00)^*#^ | 2 (0.00, 2.00) |
| Poor appetite | 26 | 1 (1.00, 1.00) | 0 (0.00, 1.00)^*^ | 1 (0.00, 1.00) | 39 | 1 (1.00, 1.00) | 0 (0.00, 1.00)^*^ | 1 (0.00, 1.00) |
| Halitosis | 22 | 1 (1.00, 2.00) | 1 (0.00, 1.00)^*^ | 1 (0.00, 1.00) | 34 | 1 (1.00, 2.00) | 0 (0.00, 1.00)^*^ | 1 (0.00, 1.00) |
| Bitter taste | 24 | 1 (1.00, 2.00) | 1 (0.00, 1.00)^*^ | 1 (0.00, 1.00) | 18 | 1 (1.00, 2.00) | 0 (0.00, 1.00)^*^ | 1 (0.00, 1.00) |
| Fatigue | 33 | 1 (1.00, 2.00) | 1 (0.00, 1.00)^*^ | 1 (0.00, 1.00) | 45 | 1 (1.00, 2.00) | 0 (0.00, 1.00)^*#^ | 1 (0.00, 1.50) |

* *P* < 0.05, compared with pre-treatment in the same group.

# *P* < 0.05, compared with the post-treatment value in the control group.
