# Supplementary material for: Efficacy and safety of adjunctive Chinese herbal decoction in treating Helicobacter pylori–positive chronic atrophic gastritis: a real-world retrospective study
Source: Front Med (Lausanne). 2026 Jan 12;12:1701915. doi: 10.3389/fmed.2025.1701915 (PMC12833376; doi:10.3389/fmed.2025.1701915)
Supplement: Supplementary file 2 [file Supplementary_file_2.docx]

Table S2.Manifestations and Diagnostic Criteria of SSDHS

| **Symptom** | **None (0)** | **Mild** | **Moderate** | **Severe** |
| --- | --- | --- | --- | --- |
| **Epigastric fullness*** | No sensation of fullness. | Occasional upper‑abdominal fullness after meals, lasting < 1 hour. | Noticeable fullness or post-meal bloating, each episode lasting 1–3 hours. | Persistent, severe fullness lasting more than 3 hours per episode. |
| **Epigastric pain*** | No pain. | Occasional pain (dull, distending, or stabbing) resolving within 1 hour. | Frequent pain (dull, distending, or stabbing), tolerable, lasting 1–3 hours. | Intense abdominal pain (dull/distending/stabbing), intolerable, lasting > 3 hour. |
| **Heaviness of limbs*** | No sensation of heaviness. | Occasional limb heaviness resolves within 1 hour. | Daily heaviness with mild impact on work, resolves in 2–3 hours. | Continuous heaviness, significantly impairs work, not relieved by rest. |
| **Loose stools*** | Normal stool consistency. | Soft or slightly loose stools, ≤ 3 bowel movements/day. | Loose stools, 4–5 bowel movements/day. | Mushy stools, > 6 bowel movements/day. |
| **Poor appetite**^#^ | Normal appetite. | Decreased interest in eating but maintains usual intake. | Little or no appetite; food intake reduced by ~ 1/3. | Anorexia; intake reduced by > 1/2. |
| **Bitter taste in mouth^#^** | No bitter or sticky taste. | Occasional bitter/sticky sensation, no impact on eating. | Frequent bitter/sticky sensation, mildly affects eating. | Persistent bitter/sticky taste, significantly impairs eating. |
| **Halitosis (bad breath)^#^** | No noticeable odor. | Subjective awareness of bad breath. | Bad breath noticeable to others. | Severe, offensively strong halitosis. |
| **Fatigue**^#^ | Normal energy level. | Mild fatigue, less talkative but able to work. | Noticeable fatigue, reduced speech and work capacity. | Extreme fatigue, strong desire to lie down; markedly impaired work ability. |

*** = Main symptom**, Scoring: None = 0; Mild = 2; Moderate = 4; Severe = 6; **# = Secondary symptom**, Scoring: None = 0; Mild = 2; Moderate = 4; Severe = 6; **Diagnostic criteria for Spleen–Stomach Damp‑Heat Syndrome:** At least **two** main symptoms (*) **and** **two** secondary symptoms (#) must be present concurrently.
